# Expression of the disease on female carriers of X-linked lysosomal disorders: a brief review

**DOI:** 10.1186/1750-1172-5-14

**Published:** 2010-05-28

**Authors:** Louise LC Pinto, Taiane A Vieira, Roberto Giugliani, Ida VD Schwartz

**Affiliations:** 1 Postgraduate Program in Child and Adolescent Health, UFRGS, Porto Alegre, Brazil; 2 Medical Genetics Service, HCPA, Porto Alegre, Brazil; 3 Postgraduate Program in Medical Sciences, UFRGS, Porto Alegre, Brazil; 4 Department of Genetics, UFRGS, Porto Alegre, Brazil

## Abstract

Most lysosomal diseases (LD) are inherited as autosomal recessive traits, but two important conditions have X-linked inheritance: Fabry disease and Mucopolysaccharidosis II (MPS II). These two diseases show a very different pattern regarding expression on heterozygotes, which does not seem to be explained by the X-inactivation mechanism only. While MPS II heterozygotes are asymptomatic in most instances, in Fabry disease most of female carriers show some disease manifestation, which is sometimes severe. It is known that there is a major difference among X-linked diseases depending on the cell autonomy of the gene product involved and, therefore, on the occurrence of cross-correction. Since lysosomal enzymes are usually secreted and uptaken by neighbor cells, the different findings between MPS II and Fabry disease heterozygotes can also be due to different efficiency of cross-correction (higher in MPS II and lower in Fabry disease). In this paper, we review these two X-linked LD in order to discuss the mechanisms that could explain the different rates of penetrance and expressivity observed in the heterozygotes; this could be helpful to better understand the expression of X-linked traits.

## Introduction

The lysosomal disorders (LD) are a heterogeneous group of approximately 50 disorders [1,2] with prevalence around 1:5,000 to 1:7,000 in live births [3,4]. Among other mechanisms involved in the degradation of macromolecules in lysosomes, the disease may be due to the deficiency of a specific hydrolase, a defect on the post-translational processing of the enzyme, or a transport defect across the lysosomal membrane [2,5]. The deficiency of a single enzyme or protein causes the blockage of an entire pathway making the substrate inaccessible to further hydrolysis by other lysosomal enzymes. It is important to stress that the gene products involved in the LD usually are not cell autonomous, as they could be secreted and uptaken on cells which do not produce them. Furthermore, in most LD more than one compound is accumulated, as in Mucopolysaccharidosis II (MPS II or Hunter syndrome), in which the main storage materials are dermatan sulphate and heparan sulphate; however, other substrates like ganglioside GM2 and GM3 and subunit c of mitochondrial ATP synthase are also accumulated in the brain [6]. The new concepts in cell biology led to the proposal of a new classification of LSD by Platt and Walkley [2] (Table 1).

**Table 1:** Classification of LSDs (adapted from Platt and Walkley, 2004 [2])

Molecular defect	Enzyme deficiency	Disease example	OMIM number
Primary lysosomal hydrolase defect	α-Galactosidase A Iduronate-sulfatase	Fabry disease MPS II	301500 309900
Post-translational processing defect of lysosomal enzymes	Multiple sulphatase deficiency (17 sulphatases)	Multiple sulphatase deficiency	272200
Trafficking defect for lysosomal enzymes	N-acetylglucosamine-1-phosphotransferase	Mucolipidosis type II	252500
Defect in lysosomal enzyme protection	β-Galactosidase and Neuraminidase deficiency	Galactosialidosis	256540
Defect insoluble non-enzymatic lysosomal proteins	Hexosaminidase activator deficiency	GM2 type AB (Tay-Sachs disease variant AB)	272750
Transmembrane (non-enzyme) protein defect	Lysosomal-associated membrane protein 2	Danon disease	300257
Unclassified	Intracellular accumulation of autofluorescent of lipopigments storage material	Neuronal ceroid lipofuscinoses (CLN4)	204300

Among the over 300 human X-linked diseases described so far, only three are LD: Fabry disease (MIM 301500), MPSII II (MIM 30900), and Danon disease (MIM 300257). This review will focus on the clinical heterogeneity found among heterozygotes of the two most frequent conditions: Fabry disease and MPS II.

### Fabry disease

Fabry disease is a rare X-linked lysosomal inborn error of glycosphingolipid catabolism which results from the deficient activity of lysosmal hidrolase α galactosidase A (α-GAL; EC 3.2.1.22). The estimated incidence of this rare disease is 1:40,000-117,000 live male births [4,7,8]. This figure, however, may be underestimated, as screening performed in newborn males in a Northwestern Italian region showed an incidence of 1 in ~4,000 males [9]. The α-galactosidase gene (*GLA *300644) is located in Xq22.1 and spans 12 kb of DNA comprising 7 exons [8]. More than 400 mutations in the *GLA *gene have been described in Fabry patients, most of them private [10-12]. The enzyme deficiency leads to a progressive accumulation of globotriaosylceramide (Gb3) and deacylated globotriaosylsphingosine (lyso-Gb3).

**Figure 1:**
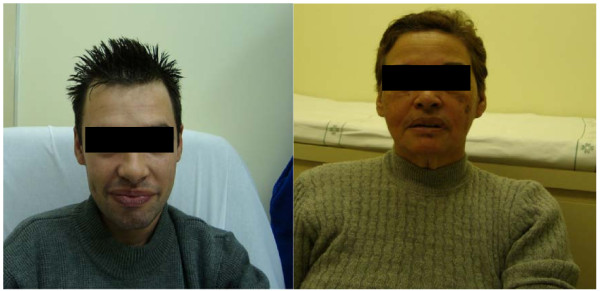
**Male patient with Fabry disease (left, child) and a heterozygote for Fabry disease (right, mother)**. The male patient is 21 years old and presents pain in hands and achroparestesias, temperature intolerance, hypohidrosis, and proteinuria. He presents a 30delG in the α-GAL gene. The mother is 62 years old and has diabetes mellitus, cardiopathy and proteinuria. She is a carrier of 30delG in the α-GAL gene. Image use authorized by patients.

Fabry disease is a multisystemic disorder that affects the vascular endothelium, renal glomeruli and tubules, dorsal root ganglia, cardiac myocytes, conduction tissue and valves, cornea and skin. Signs and symptoms include: angiokeratoma, progressive renal impairment, proteinuria, acropharestesia of hands and feet, cardiac hypertrophy and conduction abnormalities, ischemic events, corneal dystrophy, hypohidrosis and impaired temperature regulation [8,11-13]. A mother and her son with Fabry disease are shown in Figure 1.

The prevalence of females carrying the Fabry mutation was estimated as 1:339,000 in the UK [14]. Heterozygote females were usually described as asymptomatic or as exhibiting intermittent and mild symptoms of the disease [7,8]. The most common signs and symptoms in Fabry disease are shown in **Table S1**, Additional file 1[8,14-19]. Deegan *et al*. [16] showed that 70% of the heterozygote females may display signs and/or symptoms. The disease in females can be as severe as in male patients [7,20,21]; however, a slow rate of progression and a high phenotypic variability is more common [14,22]. Life expectancy is reduced in about 20 years in heterozygous females [23].

The measurement of the α-GAL activity does not always allow the identification of the heterozygotes [8,23], and it is believed that there is no correlation between α-GAL activity (in plasma or leukocytes), genotype and the severity of the clinical picture presented by heterozygotes [2]. However, some authors stated that females with classic Fabry disease usually have lower α-GAL activity and increased urinary Gb3 levels [24]. On women suspected of presenting Fabry disease due to symptomatology or findings on a tissue biopsy but with a negative family history, DNA analysis is often required for the definitive diagnosis [15,25]. On the other hand, Wilcox et al. [18] showed that leukocyte α-GAL activity can point to diagnosis in 88% of females; this suggests that used in conjunction with urinary Gb3 measurement [26] this test may be an efficient way to screen females at high risk to be carriers.

### Mucopolysaccharidosis type II

The incidence of MPS II is estimated between 1:110,000 and 132,500 live births [27,28]. MPS II is an X-linked recessive inborn error of metabolism that results from the deficiency of lysosomal iduronate-2-sulfatase (IDS; EC 3.1.6.13). The impaired IDS function leads to the storage of glycosaminoglicans (GAGs) in many organs and tissues. The partially degraded GAGs are heparan sulfate and dermatan sulfate, which are present in increased concentrations in the urine of patients. The *IDS *gene was mapped to Xq28 and contains 9 exons that spread over 24 kb [29,30]. More than 300 mutations have been described [10]. And there is also a pseudogene located 20 kb from the active gene [31].

**Figure 2:**
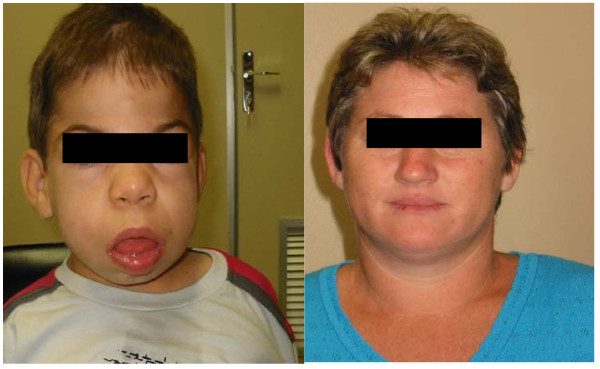
**Male patient with MPS II (left, child) and a heterozygote for MPS II (left, mother)**. The patient is 10 years old and presents severe mental handicap, coarse face, hepatosplenomeagly, dysostosis multiplex, joint contractures, obstructive airway disease, mitral regurgitation, deafness, and hydrocephaly. He presents a total deletion of the *IDS *gene. The mother is a MPS II carrier and is asymptomatic. Image use authorized by patient.

MPS II is also a multisystemic disorder, which includes the following main manifestations: coarse facies, short stature, hepatosplenomegaly, dysostosis multiplex, joint contractures, obstructive airway disease, deafness, hydrocephaly, recurrent sinopulmonary infections, valve disease and, in some patients, mental impairment [32]. The disease is classified as mild or severe according to the absence or presence of mental retardation [33,34]. MPS II is chronic, progressive, and, in its severe form, death can occur as early as at the age of 15. Cardiac and pulmonary diseases are the most common cause of death [32]. A heterozygote mother and her child affected with MPS II are shown in Figure 2.

In MPS II, the clinical disease in females is very rare, and most of the cases reported in the literature have the severe phenotype [35-43] (**Table S2**, Additional file 2). At least ten female cases have already been described [43].

In most cases, the identification of heterozygote females with MPS II by the measurement of the IDS activity is not reliable [32,44,45]. It is suggested that the lower the value of IDS, the higher the probability that these women will be a heterozygote [32]. Unfortunately, GAGs measurement in the urine is not helpful to identify carriers either [46]. Usually, the only way to reach the definitive diagnosis is through genetic analysis [47].

## X-Linked Inheritance

X-linked inheritance was first described by Morgan [48,49], and colorblindness was one of the first diseases to be recognized as having this pattern of inheritance [48]. The classic X-linked disorder usually shows a vertical transmission in which heterozygote females transmit the allele down to her sons and daughters; daughters of affected males are always heterozygotes; sons of affected males are always normal. Traditionally, these diseases are described as dominant if they are expressed both in heterozygotes and hemizygotes, or as recessive if they are expressed predominantly or almost exclusively in males. A subgroup of X-linked dominant disorders includes those characterized by male lethality or reduced male-viability (X-linked dominant male-lethal disorders) while semidominant X-linked diseases would occur when the heterozygotes present a phenotype intermediate to the phenotype of affected hemyzygotes and normal homozygotes [50].

It is true that the vast majority of mutations in X-linked genes will lead to diseases only in males [51]. However, many of the X-linked diseases show different rates of penetrance and expressivity in both genders. The following situations can be observed: 1) for some conditions, such as Fabry disease, heterozygotes are usually affected but tend to have a milder and more variable phenotype than hemizygotes (e.g., semidominant inheritance); 2) for other diseases, as MPS II, penetrance in heterozygotes is very low and, consequently, very few heterozygotes are affected (e.g., recessive inheritance); 3) on the other hand, as in Vitamin D resistant rickets, penetrance and severity are high in both genders (e.g., dominant inheritance); 4) finally, as in Rett syndrome, penetrance is high in both genders, but males usually die very early (intrauterus), and only females are usually affected at birth (e.g., lethal dominant inheritance) [21,48,52].

### Classification of X-linked disorders

According to Dobyns *et al*. [48], the traditional classification of X-linked diseases in dominant or recessive should not be used for humans. The authors made this statement because this classification was originally based on the *Drosophila *model, and some concepts cannot be applied to humans, since there are differences in sex determination and dosage compensation for genes on the X chromosome between the two species (Table 2). The mechanism that is responsible for dosage compensation in *Drosophila *is based on the X transcription speed, which is higher in males, and not in X-inactivation. Therefore, a *Drosophila *female that is heterozygous for a null mutation in an X-linked gene will not present cell mosaicism as it would be expected in female human cells. In other words, as the concept of dominance and recessivity depends on the expression on the two alleles inside the cell, it would not be applied to X-linked diseases, as in most of these conditions only one allele is expressed in the cell due to the X-inactivation mechanism [53].

**Table 2:** Comparison between *Drosophila *and humans regarding the compensation-dose mechanism (based on Dobyns *et al*., 2004 [48])

Characteristics	*Drosophila*	Human
Female	6A+XX	46, XX
Male	6A+XY	46, XY
Fertile	6A+XX and 6A+XY	46, XX and XY
Infertile	6A+XO	45, X
Dosage compensation in X chromosome	Transcribing rate of the X chromosome	X-inactivation
X- inactivation	No	Yes

Based on a literature review, Dobyns *et al*. [48] classified 32 X-linked diseases according to penetrance (high, intermediate, and low) and severity index (proportion of all symptomatic individuals with a phenotype classified as severe among all symptomatic carriers) both for males and females. Fabry disease was classified as presenting high penetrance (100%) and high severity index (84%) for males; for females, however, the penetrance was high (70%) but the severity index was not (4%). For MPS II, males were also found to present high penetrance (100%) and severity index (81%); females, however, presented both low penetrance and low severity index rate. This low severity index rate for MPS II females [48] could be contested, as the majority of the patients described presented the severe form of the disease (**Table S2**, Additional file 2). Due to the clinical heterogeneity found, these authors suggested that X-linked disorders should be analysed taking into account biological mechanisms other than X-inactivation, such as the cell-autonomy of the gene product and its early toxicity to the cell (Table 3). In the lethal X-linked dominant diseases, the gene product would be detrimental to the cell at very early stages and, as a consequence, the viability of the affected males would be reduced; expressivity would be variable in heterozygotes, e.g., a combined effect of both the X-inativaction pattern and positive and negative selection mechanisms [50].

**Table 3:** Relation between genic product properties, cellular selection, X-inactivation and phenotype of heterozygotes for X-linked diseases: the example of Fabry disease and MPS II (based on Dobyns *et al*., 2004 [48] and Dobyns, 2006 [53])

Type of gene product	Positive cellular selection (gene product causing early cell death)	Clinical Phenotype in Heterozygotes	Probably X-inactivation Pattern	Disease
Cell-autonomous	No	Normal	Random or skewed favorable to the normal allele	Fabry disease^3^
	No	Abnormal	Random or skewed unfavorable to the normal allele^4^	Fabry disease^3^
Non-cell-autonomous^1 ^or functionally non-autonomous^2^	No	Normal	Random or skewed favorable to the normal allele	MPS II
	No	Abnormal	Skewed favorable to the abnormal allele	MPS II

^1^Non-autonomous gene products would include primarily secreted proteins; ^2^Functionally non-autonomous would include proteins expressed in non-clonal, multinucleated cells and small molecules that pass freely between cells; ^3^Athough the genic product of Fabry disease is non-cell autonomous, the hypothesis is that cross-correction capacity in this disease is low and, as a result, it is being classified in this table as cell-autonomous.^4^The phenotype can be modulated by the presence of a randomic X-inactivation, e.g., the phenotype is abnormal, and/or by an unfavourable skewed to the normal allele; then, a more severe phentoype is expected.

## Twinning and X-Linked Diseases

Discordant phenotypes in monozygous twin females (MZF), who were heterozygous for X-linked disorders, have already been reported for many diseases, including Duchenne Muscular Dystrophy (DMD), G6PD deficiency, Haemophilia B, Fragile-X syndrome, Green color deficiency, MPS II and Fabry disease [39,54-60]. Clinical expression of the X-linked diseases in one of the MZF seems to be proportionally more frequent than the expression in heterozygote females. Also, it seems that concordant expression between MZF heterozygotes has never been reported [60].

Three female Fabry twin pairs with discordant phenotype were described in the literature. Only one MZF pair was confirmed regarding monozigosity. At the age of 26 years, the affected MZF presented severe signs and symptoms: renal proteinuria, angiokeratotic lesions in the pelvic region and thighs, verticillate cornea, and acroparesthesia in all four limbs. Both MZF were heterozygous for the G10182A mutation (exon 5). The phenotype was attributed to the skewed X-inactivation favoring the *GLA *allele mutant in the affected MZF, showing an opposite direction in the normal MZF [54-56]. For female MZF MPS II twins, only one case has been described in the literature, and the affected twin had a normal karyotype and skewed X-inactivation also favoring the *IDS *mutant allele [39].

Tiberio [60] reviewed some characteristics common to the X-linked diseases in MZF as follows: 1) karyotypes are normal; 2) one twin is severely affected and the other is spared; 3) intermediate phenotypes have not been described; 4) the X-inactivation pattern is opposite to skewing (abnormal allele inactivated in most cells of the normal twin, and the normal allele inactivated in most cells of the affected twin), and 5) the disease maps into regions Xq27-28, Xp21, Xq22 and Xp22. Due to these observations three hypotheses came up to explain the discordant phenotype in these cases: 1) casual aggregation of cells carrying the same inactive chromosome that predisposes twining [61]; 2) a "sampling effect" through early symmetric or asymmetric splitting of the inner cell mass occurring after the X-inactivation [61,62], and 3) early mitotic crossing over, segregation of one crossed and one uncrossed chromatid in the daughter's cells, and obligate inactivation of the uncrossed chromatid [63]. Unfortunately, it seems that none of these hypotheses can completely explain the MFZ discordant for X-linked diseases [60].

The X-inactivation occurs very early in embryonic life in the late blastocyst stage of embryogenesis [41,63], and one possible explanation of the variable expression of an X-linked abnormality in MZF lies on the fact that X-inactivation precedes the twinning event [56,57,61,64-66]. It was also observed that in MZF who are dichorionic, twinning occurs prior or around the onset of X-inactivation and, in monochorionic twins, twinning occurs later [67]. So it might be expected that discordant phenotype should be more frequent in twins dichorionic; however, anedoctal cases of discordant X-linked diseases in dichorionic MZF have already been reported [39,58]. A new explanation proposed by Monteiro *et al*. [65] is that monochorionic MZF is, in fact, a heterogeneous group that differs in the timing of the twinning event after the onset of X-inactivation [59].

There are many other possible explanations for discordant phenotypes between monozygotic twins: environmental causes, several embryologic mechanisms, vascular abnormalities, defect of midline structures and postzygotic changes in the genetic material, including somatic mutations [62].

## X-Inactivation, Fabry Disease and MPS II

The main question to be answered is if skewed X-inactivation is the main mechanism responsible for the different rates of penetrance and expressivity presented by heterozygotes for Fabry and MPS II diseases (Table 3).

### X-inactivation process

The X-inactivation was first described by Lyon [68,69], who suggested that the X-inactivation was based on the pattern of patches in heterozygote female mice. This author proposed some general rules for X-inactivation: 1) normal females have just one active X chromosome; 2) X-inactivation occurs early in development; 3) the inactive X can be maternal or paternal, and the choice is random, and d) X-inactivation is irreversible in somatic cells and through all its descendants. Because females have two X chromosomes and males only one, the X-inactivation provides a mechanism called "compensation dose". As a result, females are mosaic [21].

The X-inactivation process is dependent on many factors and is tissue specific [52]. It is initiated on the X-inactivation center (Xic) located at Xq13.2 [70]. The X-inactivation specific transcript (*Xist*; MIM 314670) gene is responsible for the process of *cis *inactivation [71], and it is transcribed only in the inactive X chromosome. In Xic, it is also transcribed, in the antisense orientation, the X-inactive specific transcript-antisense (*Tsix*; MIM 300181) that has the function of regulating the early expression and the choice of which X chromosome will be inactivated [72]. As the X-inactivation pattern is established, all descendents cells will have the same pattern. X-inactivation in somatic cells is very stable [73], permanent and irreversible, except in oogenesis and in spermatogenesis [74]. The main steps of inactivation are: counting, choice and initiation [75]. Actually, the first step is to maintain one of the active X chromosomes [21], and this is attributed to the existence of an autosomal limited factor [73]. Many epigenetics modifications are necessary to guarantee the maintenance and the herdability of the X-inactivation [76]. However, not all genes present in the X chromosome are inactive, and it is estimated that about 25% of the genes [77] escape from the inactivation, most of them located in the X chromosome short arm.

Lyonization follows the Gaussian distribution in the female population, and it is expected that females would be 50:50 for genes that underwent X-inactivation, but ratios 60:40 or 70:30 are the most common [78], and even 80:20 or 90:10 can be considered normal [51,67]. However, it was shown that at least 5-10% of normal females present extreme skewing of X-inactivation [79], while some studies showed a range between 4% and 33% [80]. Extreme skewed X- inactivation is defined as affecting more than 90% of one allele [81] and may be a consequence of the following: 1) biases in the choice of which X chromosome to inactivate; 2) X chromosome mutations or rearrangements which affect the viability of cells with one or the other active X, or 3) stochastic factors [73,78]. Highly skewed X-inactivation, defined as more than 80:20, could be pathogenic [82].

Studies in peripheral blood cells show that skewing of inactivation increases with age, but the practical significance of this finding is uncertain. However, it is possible that skewing in an advanced age is correlated with the presence of a pathogenic allele in the X chromosome [78]. The age-skewing process can be caused by stochastic clonal loss of hematopoietic cells [83] or the competitive advantage for hematopoietic stem cells with a specific genotype of X-linked genes [84].

### Skewed X-inactivation and diseases

Traditionally, the main mechanisms that are said to influence the expression of the X-linked disorders are: skewed inactivation [43,69,85,86], clonal expansion, and somatic mosaicism [87]. Therefore, heterozygotes for X-linked diseases could be symptomatic if: 1) the mutation confers a proliferative advantage to the mutant cells, OR 2) there is skewed X-inactivation due to any reason (X/autosomal translocation, mutation in the *XIST *gene, etc), and the mutant is in the active X chromosome [36,43,87]. However, there are other explanations for the heterozygotes for an X-linked disease presenting with signs and symptoms, such as the occurrence of hemizygozity for the mutant allele (e.g., the patient is a carrier of the mutation but full X monosomy is present); the occurrence of uniparental disomy for the mutant X chromosome, and the absence (or deficiency) of cross-correction [39]. Structural rearrangements involving the X chromosome, as X/autosomal translocations and deletions, could disrupt the normal allele and may cause loss of heterozygozity or contiguous gene syndromes. Several diseases, such as DMD, had their genes mapped to the X chromosome after patients with rearrangements involving the same X chromosome region were identified. In the case of MPS II, patients presenting deletions involving not only the *IDS *gene but also the *FMR1 *(involved in the X-fragile syndrome) have been described [88].

As previously mentioned, skewed X-inactivation has been shown to be responsible for clinical manifestations in female carriers of X-linked diseases, such as DMD [57]. Skewed X-inactivation also seems to be involved in other situations such as recurrent pregnancy loss [89,90] and breast cancer [91]. Deviation from the random X-inactivation can be primary or secondary. Primary as a result of the deviation of X-inactivation itself, e.g., a mutation in *XIST *gene, or secondary if the non-random X-inactivation has a consequence like cell selection [22,92].

It is possible to identify four X-inactivation patterns in heterozygotes for X-linked diseases as follows: 1) random inactivation is usually associated with a normal phenotype; 2) random inactivation leads to manifestations in a continuum spectrum, and a normal phenotype requires skewing, favoring the normal allele in the expressing tissue; 3) always extreme skewing, as mutant cells die or fail to develop, or migrate to destination; 4) gradual skewing because of cell selection due to a proliferative advantage of wild type (or mutant) cells expressing tissue [21].

Unbalanced X-inactivation can occur as a stochastic variation or due to genetic factors influencing the X-inactivation itself or the postinactivation selection mechanism [22]. The first two are rare and occur independent of the kind of mutation in the gene of the disease [22,67].

## Fabry Disease: Explanations for the Female Phenotype

Symptomatic heterozygous females for Fabry disease can be as frequent as 60-70% [22,93]. Sometimes, symptoms are as severe as in males [7,93]. It has been suggested that X inactivation studies could be helpful in predicting the female phenotype in Fabry disease [11,22]. For some authors [8,15,23] skewed X-inactivation plays an essential role in the phenotype expression in heterozygotes of Fabry disease. Dobrovolny et al. [11] showed that some females with preferential X-inactivation have more rapid disease progression and suggested that X-inactivation is the major factor for determining the severity of clinical involvement and morbidity in Fabry heterozygotes.

However, for other authors [52], the phenotype was not due to skewed X-inactivation, and severity was not correlated with deviation either. They concluded that the X-inactivation in leukocytes in females with Fabry disease is not useful to predict prognostic and should not be used to define therapeutic options. And disease progression was related to a cross-correction mechanism which would have a decreasing efficiency throughout the years, which would explain the fact that heterozygote females for Fabry disease get more symptomatic in time [22].

The cross-correction mechanism is also called metabolic cooperation. Lysosomal enzymes freely enter and leave lysosomes and are transferred from one cell to another by manose-6-phosphate-mediated endocytosis. When the enzyme is deficient, the non-digested products accumulate in the lysosome [87]. In Fabry disease, either the amount of active enzyme secreted may be insufficient, or the secreted enzyme is not adequately taken up by normal cells, and this is not enough to cross-correct the deficient synthesis of the abnormal cells [16,21,87]. This last conclusion was suggested by the fact that the activity of the enzyme in plasma or leukocytes may not reflect the situation within the lysosome of relevant cell types of Fabry disease, which suggests that the uptake by normal cells may be defective [12,16]. It seems that a distinct pattern of recaptation due to the mannose-6-phosphate receptors and the localization in the enzyme can be responsible for a lower-uptake enzyme, and, as a consequence, some females will manifest the disease [87]. In the case of Pompe disease (α-glucosidase deficiency) a LD of autosomal recessive inheritance, abnormalities in the trafficking of intracellular mannose-6-phosphate receptors have been described. This would be the result of abnormal substrate storage, with impact on the internalization of the endogenous enzymes [94]. According to Dobyns *et al*.[48] cross-correction ability in Fabry disease would be largely restricted, as normal heterozygotes could present skewed X-inactivation in favour of the normal allele, and symptomatic heterozygotes may show random X-inactivation (Table 3). Another hypothesis is related to a new concept ("cross-induction"), for increased levels of plasma lyso-Gb3 (a deacylated form of Gb3) were found in symptomatic heterozygotes for Fabry disease and appear to be positively correlated with the severity of the clinical picture [95]. If this is true, perhaps lyso-Gb3 is widely diffusible and has the capacity to inhibit α-GAL activity produced by cells which have an active non-mutated X-chromosome leading, therefore, to clinical symptomatology in most heterozygotes.

The following other factors affect expression in Fabry disease: 1) the nature of the mutation - and correlation with enzyme activity [15], and 2) the blood group (patients of blood groups B and AB are more severely affected than individuals of other groups [8], probably because these patients accumulate, in addition to globotriaosylceramie and galabiosylceramide, two further glycosphingolipids [15]).

### MPS II Disease: explanations for female phenotype

Different from Fabry carriers, heterozygote MPS II females are spared from the disease. But why? It may be attributed to the cross-correction of cells (in which the active X is the one with the mutation) by the functional enzyme secreted by cells in which the non-mutant gene is active [96]. Schwartz *et al*. [97] suggested that in some cells of MPS II carriers, like chondrocytes or hepatocytes, the X chromosome carrying the mutant allele would be preferentially inactivated or reduce cell viability. Consequently, MPS II heterozygous females are rarely affected unless there is a simultaneous presence of two mutant alleles or if a coincidental genetic defect happens, leading to skewed X-inactivation or hemizygozity in heterozygotes (Table 3). According to Dobyns *et al*. [48] as the cross-correction ability would be preserved in MPS II, the clinically normal heterozygotes would present randomic X-inactivation or skewed X-inactivation with predominant expression of the normal X chromossome, while the symptomatic heterozygotes would present skewed X-inactivation with predominant expression of the mutated allele (Table 3). These findings are in accordance with the literature, as shown in **Table S2**, Additional file 2.

### Concluding remarks and the future

Most females are mosaics and have a mixture of cells expressing either their mother's or father's X-linked genes. Often, cell mosaicism is advantageous, for it ameliorates the deleterious effects of X-linked mutations and contributes to physiological diversity. Although sometimes mosaicism brings a significant biological advantage, the outcome is never certain [89].

It is interesting that, although Fabry disease and MPS II are both X-linked LD, they show many differences in clinical expression of heterozygous females. The main differences, at the moment, could be explained by: 1) cross-correction or cross-inducing mechanisms; 2) the skewed X-inactivation [21].

As proposed by Dobyns *et al*. [48], the X-linked diseases show a continuum in penetrance, and new rules must be established to explain the various phenotypes observed. Despite the fact that over 30 years have passed since the discovery of the existence of the X-inactivation mechanism, we still have a lot to learn, and new information about cell biology is still needed to allow us to provide a more precise genetic counseling to affected families.

## Consent

Written informed consent was obtained from the patient for publication and images. A copy of the written consent is available for review by the Editor-in-Chief of this journal.

## Competing interests

The authors declare that they have no competing interests.

## Authors' contributions

All authors contributed to this review. They read and approved the final version of the article

## Supplementary Material

Additional file 1 **Table S1**. Signs and symptoms in Fabry heterozygotes: review of the literature Click here for file

Additional file 2 **Table S2**. Female patients with Hunter syndrome (based on Tuschl *et al*. 2004 [43]) Click here for file
