# Supplementary material for: Expression of the disease on female carriers of X-linked lysosomal disorders: a brief review
Source: Orphanet J Rare Dis. 2010 May 28;5:14. doi: 10.1186/1750-1172-5-14 (PMC2889886; doi:10.1186/1750-1172-5-14)
Supplement: Additional file 1 — Table S1. Signs and symptoms in Fabry heterozygotes: review of the literature [file 1750-1172-5-14-S1.DOC]

Table S1. Signs and symptoms in Fabry heterozygotes: review of the literature

| Report | N | X-  inactivation | Age  (years) | Neuropathic  Pain  (%) | Angiokeratomas  (%) | Proteinuria  (%) | Cardiac  Abnormalities  (%) | Verticillate  Cornea  (%) | Isquemic  Events  (%) |
| --- | --- | --- | --- | --- | --- | --- | --- | --- | --- |
| Desnick  *et al*. 2001 [8] | 122 | NI | 1-85 | <10 | 30 | <1*** | <1 | 70 | <1 |
| MacDermot  *et al.* 2001 [14] | 60 | NI | >18 | 70 | 35 | 31.5 | 90 | NI | 6.7 |
| Whybra  *et al.* 2001 [15] | 20 | NI | 20 | 90 | 55 | 55 | 55 | 70 | 75 † |
| Deegan  *et al.* 2005 [16] | 248 | NI | 38.2+/-18 | 77 | 40 | 35 | 59 | 40 | 7 |
| Nagasaki  *et al.* 2007 [17] | 3 | NI | N | 100 | 100 | 66,6 | NI | NI | 0 |
| Wilcox  *et al,* 2008 [18] | 1077 | NI | 0->50 | 43.3 | 17.8 | 10.6 | 10 | 12.5* | 4.2 |
| Kobayashi  *et al.* 2008 [19] | 36 | NI | 4-68 | 50 | 5.6 | 38.9 | 38.9** | 50* | 8.3 |

*NI= not informed;*Non specified ocular abnormalities **left ventricular hypertrophy *** renal failure; † MRI was done in 4/20 women*
