# Supplementary material for: Expression of the disease on female carriers of X-linked lysosomal disorders: a brief review
Source: Orphanet J Rare Dis. 2010 May 28;5:14. doi: 10.1186/1750-1172-5-14 (PMC2889886; doi:10.1186/1750-1172-5-14)
Supplement: Additional file 2 — Table S2. Female patients with Hunter syndrome (based on Tuschl et al. 2004 [43]) [file 1750-1172-5-14-S2.DOC]

Table S2 – Female patients with Hunter syndrome (based on Tuschl *et al.* 2004 [43])

| Reference | X-inactivation | Karyotype | Mutation | Twins | Form |
| --- | --- | --- | --- | --- | --- |
| Neufeld  *et al*. 1977 [35] | Not  Tested | 46,XX | Nd* | No | Neuronopathic |
| Mossmann  *et al.* 1983 [36] | Skewed^2^ | 46,XX  t(X:5)** | Nd* | No | Neuronopathic |
| Broadhead  *et al*. 1986 [37] | Skewed^2^ | delXq25 | Nd* | No | Neuronopathic |
| Clarke  *et al.* 1991 [38] | Skewed^2^ | delXq27-28 | 3-5cM-deletion | No | Neuronopathic |
| Winchester  *et al.* 1992 [39] | Skewed^2^ | 46,XX | Nd* | Yes | Neuronopathic*** |
| Sukegawa  *et al.* 1997 [40] | Skewed^2^ | 46,XX | p.R468Q/N | No | Neuronopathic |
| Sukegawa  *et al.* 1998 [41] | Skewed^2^ | 46,XX | p.R468L/N | No | Neuronopathic |
| Cudry *et al.*  2000 (case a) [42] | Skewed^2^ | 46,XX | p.L4IP/ L4IP | No | Non-Neuronopathic |
| Cudry *et al.*  2000 (case b) [42] | Skewed ^2^ | 46,XX | [g.17419del3254+  g.17418ins20]/N | No | Neuronopathic |
| Tuschl  *et al.* 2004 [43] | Skewed^2^ | 46,XX | [c.706_719del+  c.705_720insG]/N | No | Neuronopathic |

*Nd* not determined; **46,XX t(X;5)(q2?7;q3?1)(q2?6;q3?2); ***She was at school at the age of eleven and had an average intellectual ability; ¹The author reported 2 cases that were initially diagnosed with MPS II, but one of them was further diagnosed with multiple sulfatase deficiency*; ^2^*Data not shown.*
